# First Report on Intensity Bioassays for Pyrethroid Resistance in *Anopheles culicifacies s*.*l* in District Dindori of Madhya Pradesh State and Districts Kanker and Bastar of Chhattisgarh State, India

**DOI:** 10.1155/2022/1595604

**Published:** 2022-09-20

**Authors:** Ashok K. Mishra, Mrigendra P. Singh, Aparup Das, Kamaraju Raghavendra

**Affiliations:** ^1^ICMR-National Institute of Research in Tribal Health (NIRTH), NIRTH Campus, Medical, P. O Garha, Jabalpur, Madhya Pradesh, India; ^2^ICMR-National Institute of Malaria Research, Sector 8, Dwarka, New Delhi, India

## Abstract

**Background:**

The major malaria vector in India is *Anopheles culicifacies*, and indoor residual spraying (IRS) and distribution of long-lasting insecticidal nets (LLINs) are the two main vector control measures in the national program. This species has shown resistance to dichlorodiphenyltrichloroethane (DDT), malathion, and synthetic pyrethroids (SP). The study was carried out in three districts, that is, Dindori of Madhya Pradesh and Kanker and Bastar from Chhattisgarh state to know the range of resistance phenotypes and to assess the strength of resistance in *An*. *culicifacies*.

**Methods:**

*An*. *culicifacies* collected from the field was tested to determine the susceptibility status to the discriminating concentration (DC) of different insecticides, that is, DDT 4.0%, malathion 5.0%, alphacypermethrin 0.05%, and deltamethrin 0.05% following the World Health Organization (WHO) procedures. Further, intensity bioassays of the resistant *An*. *culicifacies* to 1X discriminating concentration (DC) of alphacypermethrin and deltamethrin were conducted by exposing 5X and 10X concentrations of 1X DC. Results are interpreted as per the WHO criterion.

**Results:**

The overall result of susceptibility status in *An*. *culicifacies* in districts Dindori, Kanker, and Bastar revealed confirmed resistance to DDT, malathion, alphacypermethrin, and deltamethrin registering mortality of 15% (10–20%), 70% (65–75%), 78.6% (77–82.5%), and 87% (84.8–91.3%), respectively. Further, in district Dindori and Baster, the intensity bioassay test at 5X DC of alphacypermethrin and deltamethrin revealed 99% and 100%, respectively, while in district Kanker, the species registered moderate resistance with 92.5% and 95% mortality, respectively, in 5X DC of alphacypermethrin and deltamethrin. However, in 10X DC, the *An*. *culicifacies* was susceptible to both pyrethroids (100%) in district Kanker.

**Conclusion:**

The results of the intensity bioassay tests with SP-resistant *An*. *culicifacies* suggested no change of insecticide is required in the ongoing intervention. However, regular monitoring of insecticide susceptibility and intensity bioassays in malaria vectors in view of continued use of these interventions may increase resistance and for implementing effective vector management strategies.

## 1. Introduction

In India, *Anopheles culicifacies s*.*l*. is the main malaria vector in rural and peri-urban areas and is responsible for about 65% of annual malaria transmission [1]. The Indian vector control program is mostly reliant on pyrethroid indoor residual spray (IRS) and long-lasting insecticide-treated bed nets (LLINs). *An*. *culicifacies* has developed resistance to insecticides of different classes such as dichlorodiphenyltrichloroethane (DDT) (organochlorine), malathion (organophosphate), and synthetic pyrethroids (SP), which are in use for vector control in India [2]. Insecticide resistance in malaria vectors is determined by insecticide susceptibility tests using the World Health Organization (WHO) test procedure prescribed discriminating concentration (DC) of insecticides that can measure the spread and the prevalence of insecticide resistance in vector population [3]. The WHO test procedure further suggests conduct the intensity assays on target vector with 5X and 10X DC to assess the strength of resistance and the operational significance of its use in vector control [3]. The information generated on the susceptibility status from the intensity bioassays will facilitate a choice to continue or change the insecticide/intervention. However, in India, the change of insecticide will be made by choice of alternative insecticide by considering the data of vector resistance studies and the observed impact of insecticide intervention on the epidemiology of the disease [4].

Therefore, the intensity bioassays with 5X and 10X higher concentrations of alphacypermethrin and deltamethrin were conducted in an area where earlier reported resistance at the DC (1X) of these pyrethroids, to assess the strength of resistance and the operational significance of its use in vector control in highly malarious areas of India, following the WHO insecticide test procedures [3].

## 2. Materials and Methods

### 2.1. Study Area

**Figure 1:**
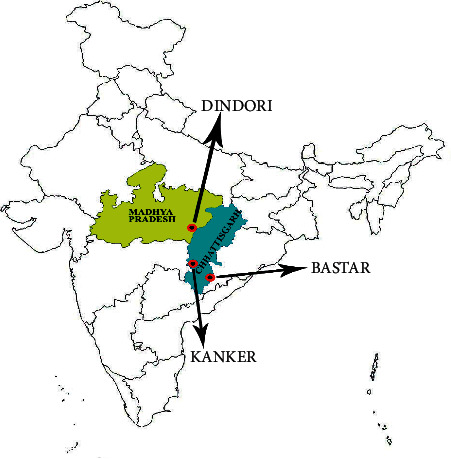
Map of India showing the location of the study district Dindori in Madhya Pradesh state and districts Kanker and Bastar in Chhattisgarh state.

The study was conducted in three highly malarious and forested districts, namely, Dindori in Madhya Pradesh (MP), and Kanker and Bastar in Chhattisgarh (CG) states of Central India (Figure 1). These districts were purposively selected as *An*. *culicifacies* reported resistance against SPs, alphacypermethrin, and deltamethrin at the DC (1X) in earlier studies. However, SP resistance was reported in this species in these states [5, 6], but the strength of resistance of these insecticides in this species was not documented so far in MP and CG states of the country.

District Dindori is located between 22.9°N and 81.08°E geo-coordinates, with total geographical area of 6432 km^2^. The average rainfall in the district was 1000–1200 mm. About 60% of the area of the district is covered with deciduous forest with largely tropical vegetation. District Dindori is one of the malarious districts in MP contributing 4% of total malaria cases in the state having 1% of the state population during 2015–2020. Annual parasite incidence (API: number of malaria positive per thousand population) of the area during 2015 to 2020 ranged from 0.1 to 6.4. LLINs were distributed in the years 2012, 2013 and 2017, and 2019 (Source: Directorate of Health Services, Govt. of MP).

Chhattisgarh is among the most malaria endemic states in India, which contributes about 18% of the annually reported malaria cases in the country in the Year 2020 with the predominance of *Plasmodium falciparum*. Districts Kanker and Bastar are located between 20.2°N–81.08°E and 19.08°N–82.02°E geo-coordinates, respectively, in the southern part of the state. The total geographical area of the districts Kanker and Bastar is 6432 km^2^ and 4030 km^2^, respectively. The average annual rainfall in the districts Kanker and Bastar is recorded between 1090–1492 mm and 1387–1621 mm, respectively. About 54% of the districts area is covered with deciduous forest with largely tropical vegetation. Kanker and Bastar are highly malarious districts with API in the range of 7.0 to 16.0 during the last 3 years preceding the survey and *P*. *falciparum* accounting ∼90% of total malaria cases (Source: Directorate of Health Services, Govt. of CG).

### 2.2. Mosquito Collection

Mosquitoes were collected from randomly selected 3 to 4 villages in the three districts where pyrethroid LLINs and DDT/pyrethroid IRS were the vector control interventions from the year 2015-16. Mosquitoes were collected from different resting sites (indoors-human dwellings/cattle sheds) in the selected villages using a mouth aspirator and flashlight during the early morning hours. The mosquitoes, after collection, were transported to the field laboratory in a cloth cage wrapped with a wet cloth. Mosquitoes were identified to species based on species-specific morphological characteristics [7]. Mixed-age-blood-fed *An*. *culicifacies* s.l. mosquitoes were identified and separated by the experienced technicians and used for insecticide susceptibility tests [8].

### 2.3. Insecticide Susceptibility Tests

The susceptibility test of *An*. *culicifacies* to WHO-prescribed DC (1X) of insecticides in district Dindori (MP) was carried out in the Year 2021, and in Kanker and Bastar (CG) in the Year 2022. Field-collected mixed-age female *An*. *culicifacies* mosquitoes were tested to determine the susceptibility status to different insecticides using impregnated papers of WHO-prescribed discriminating dosages procured from Vector Control Research Unit, University Sains Malaysia (VCRU, USM) following WHO method and kit [3]. Female mosquitoes were exposed in 4 to 7 replicates, with 15 to 20 mosquitoes in each replicate to DDT 4.0%, malathion 5.0%, deltamethrin 0.05%, and alphacypermethrin 0.05%, and respective insecticide class controls for one hour and held for 24 hours of holding period. Mosquitoes during 1-h exposure to insecticide and 24-h holding period postexposure were kept in cartons with wet towels at the bottom and calibrated with a thermo-hygrometer to maintain the ambient temperature of 25 ± 2°C and relative humidity (RH) of 80 ± 10% in the carton. After the 24-h holding period, mosquitoes were scored as dead and alive mosquitoes based on the WHO classification [3]. Percent mortality was calculated separately for the test and control replicates using the following formula:

(1) $$\begin{array}{c} \text{Observed mortality}=\frac{\text{Total number of dead mosquitoes }}{\text{Total sample size}}\times 100. \end{array}$$

If the mortality in control replicates was between 5% and 20%, the test mortality was corrected with the mortality in control replicates using Abbott's formula. In case, the mortality in the control replicates exceeds 20%, and the test was discarded.

(2) $$\begin{array}{c} \text{Corrected mortality}=\frac{\left(\text{\% observed mortality}-\text{\% control mortality}\right)}{\left(100-\text{\%control mortality}\right)}\times 100. \end{array}$$

Mortality in the replicates to the discriminating dosage of a given insecticide in the range of 98 to 100% is designated as “susceptible,” <90% as “confirmed resistance,” and mortality in the range of 90 to 97% was designated as “possible resistance” [3].

### 2.4. Intensity Bioassay Tests

Intensity bioassay tests in districts Dindori (MP), Kanker, and Bastar (CG) were carried out in the Year 2022. Exposures of insecticide-resistant mosquitoes to higher concentrations provide information on the intensity of resistance or “strength” of a resistance phenotype determined in susceptibility tests with DC (1X) [3]. The resistant population to WHO-prescribed DC is further tested for resistance intensity by exposing additional mosquito samples to 5X and 10X of the DC (1X) of the insecticides to assess the operational significance of use of that insecticide [3]. Mortality rates in insecticide intensity assays are interpreted as follows:

- Mortality in the range of 98–100% at 5X concentration indicates a low resistance intensity, and further testing at 10X concentration is not necessary and suggests continuing the existing insecticide for vector control intervention.
- Mortality of <98% at the 5X concentration indicates a moderate resistance intensity. It is recommended to assay further to the 10X concentration.
- Mortality of <98% at the 5X concentration and mortality in the range of 98%–100% at 10X concentration confirm a moderate resistance intensity.
- Mortality of <98% at the 10X concentration indicates a high resistance intensity.

If the mortality to 5X of the DC is in the range of 98 to 100%, no change in insecticide for vector control is suggested. If resistance is confirmed at 5X and especially at 10X concentrations, operational failure is likely and a change of insecticide is preferred. Additionally, the distribution of resistance should be investigated to identify resistance foci where it is most intensively expressed [3].

### 2.5. Data Analysis

The mortality data of discrimination dosage (1X) and the mortalities in intensity bioassay data obtained from the 5X and 10X of the DC were subjected to logistic regression analysis with 1X DC as reference to the 5X and 10X to find out the significance of the enhanced susceptibility.

## 3. Results

### 3.1. Insecticide Susceptibility Tests

**Figure 2:**
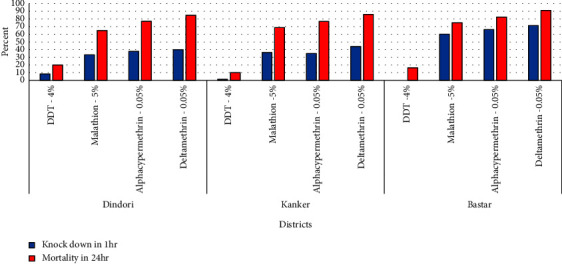
Phenotype insecticide susceptibility status of An. culicifacies s.l. to discriminatory dosages of different insecticides in district Dindori (Madhya Pradesh state) and districts Kanker and Bastar in Chhattisgarh states during 2021-2022.

The overall result of susceptibility status in *An*. *culicifacies* in districts Dindori, Kanker, and Bastar revealed confirmed resistance to DDT, malathion, alphacypermethrin, and deltamethrin registering mortality of 15%, 70%, 78.6%, and 87%, respectively. Furthermore, the district-wise analysis of the susceptibility status showed confirmed resistance to DDT (mortality—20%), malathion (mortality—65%), alphacypermethrin (mortality—77.1%), and deltamethrin (mortality—84.8%) in district Dindori; confirmed resistance to DDT (mortality—10%), malathion (mortality—68.8%), alphacypermethrin (mortality—77%), and deltamethrin (mortality—86%) in district Kanker; and confirmed resistant to DDT (mortality—16.3%), malathion (mortality—75%), and alphacypermethrin (mortality—82.5%), but was in possible resistance category to deltamethrin (mortality—91.3%) in district Bastar (Figure 2).

### 3.2. Intensity Bioassay Tests

Alphacypermethrin- and deltamethrin-resistant *An*. *culicifacies* in adult susceptibility tests in Dindori were exposed to intensity bioassays in March 2022 to 5X (0.25%) and to 10X (0.5%) concentration of both the insecticides and the species registered >99% mortality, and the species was susceptible (Table 1) and was in low resistance category, and further testing with 10X concentration was not suggested and the insecticide in use in ongoing vector control intervention can be continued [3].

Alphacypermethrin- and deltamethrin-resistant *An*. *culicifacies* in Kanker to 1X concentration (0.05%) registered 92.5% and 95.0% mortality in intensity bioassays with 5X concentration (0.25%) and registered moderate resistance to both the SP insecticides and intensity bioassays with 10X (0.5%) are done. However, the species was completely susceptible to 10X concentration (0.5%) of both the insecticides (Table 1). The results in Kanker suggest no change in insecticide, and the insecticide in the ongoing vector control intervention can be continued [3].

In Bastar district, *An*. *culicifacies* was susceptible (99–100%) to 5X concentrations of alphacypermethrin and deltamethrin (Table 1). Further testing with 10X concentration of the insecticides is not suggested. Thus, as per the criterion no need of change of insecticide is needed [3].

Logistic regression analysis of the mortality data on bioassays with 1X (0.05%) DC and the 5X (0.25%) and 10 X (0.5%) DC revealed that alphacypermethrin was likely to be 29.33, 3.68, and 16.76 times more susceptible to 5X concentration than 1X DC in districts Dindori, Kanker, and Bastar, respectively, for 1-h exposure to the insecticide and after 24 h of holding. Further analysis showed that in exposure to alphacypermethrin, 10X concentration compared with 1X DC was 100% susceptible as all the *An*. *culicifacies* died at 10X concentration of alphacypermethrin in districts Dindori, Kanker, and Bastar (Table 2).

Similarly, at 24-h observation to deltamethrin was also likely to be 3 times more susceptible in 5X concentration than 1X DC in district Kanker but was not found significant statistically (*p*=0.148). However, in the districts Dindori and Bastar, all the *An*. *culicifacies* died at 5X and 10X concentrations, and in the district Kanker, all the *An*. *culicifacies* died at 10X concentration of deltamethrin (Table 2).

At 24 h, the mortality of *An*. *culicifacies* at 5X concentration of alphacypermethrin in district Dindori was 99%, which was significantly higher than district Kanker (chi-square = 4.35; *p*=0.037). District Bastar also showed higher mortality (98.75%) than district Kanker, but the difference was not found significant (chi-square = 3.23; *p*=0.072), whereas mortality in deltamethrin was 100% in districts Dindori and Bastar in comparison with the district Kanker (95%) and the difference was significant statistically (*p* < 0.05).

Further comparison of mortality between 5X and 10X concentrations of alphacypermethrin and deltamethrin in district Kanker did not show any significant differences (*P* > 0.5). The statistical analysis indicated *An*. *culicifacies* registered moderate resistance in resistance intensity assays in districts Dindori and Kanker and was susceptible in Bastar suggesting no change in ongoing vector control strategy.

## 4. Discussion

The decrease in malaria burden in India is mainly due to the continuous use of SP insecticide-based vector control methods [9]. Pyrethroid resistance in this primary malaria vector *An*. *culicifacies* was reported in many districts of India including in the present three study districts [2, 5, 6]. A study in CG state reported *An*. *culicifacies* susceptible to alphacypermethrin in district Kanker during years 2006–2007 [10], but in later surveys in the Year 2009, the species was reported pyrethroid resistant [6]. A study in the years 2009 and 2010 reported multiple resistance to DDT, malathion, and deltamethrin in *An*. *culicifacies* from different districts of CG state including the two districts of the present study [2], Kanker, and Bastar [6], and to deltamethrin in district Bastar in the year 2014 and 2015 [11]. In a study in MP during 2009–2010 in nine tribal districts, *An*. *culicifacies* registered multiple resistance to DDT and malathion and mostly in the verification required category to deltamethrin, but this species was reported resistant to deltamethrin in the district Dindori of the present study [5]. Thus, *An*. *culicifacies* was reported resistant to pyrethroids in the three study districts since more than a decade. In the present study, the species reported confirmed resistance to 1X DC of alphacypermethrin and deltamethrin in the study districts, Kanker (CG) and Dindori (MP), while in Bastar (CG), it was confirmed resistant to alphacypermethrin and in possible resistance category to deltamethrin.

The intensity bioassays with resistant *An*. *culicifacies* registered moderate resistance with 5X DC of alphacypermethrin (mortality—92.5%) and deltamethrin (mortality—95%) in district Kanker but were completely susceptible to 10X concentration. In contrast, the species was susceptible to 5X concentration of both the insecticides in two other districts, Dindori and Bastar, that is, mortality in the range of 98–100% (Table 1). The results suggested no change in insecticide in the ongoing vector control interventions in the all the three study districts. In a maiden study in India, a study in ten malaria endemic districts of southern Odisha with confirmed pyrethroid resistance with mortality in the range of 70 to 80% to WHO-prescribed DC of deltamethrin 0.05% (1X) and registered increased susceptibility to deltamethrin 0.25% (5X) of <98% (range: 92–97%) indicating moderate resistance but registered complete susceptibility to deltamethrin 0.5% (10X) prompting no change in use of ongoing use of SP in LLINs for vector control [12]. Similarly, in the present study, all three studied districts of MP and CG registered mortality in the range of 77–82% to WHO-prescribed DC of alphacypermethrin 0.05% (1X) and 85 to 91% to deltamethrin 0.05% (1X) and were relatively more susceptible than in Odisha population. *An*. *culicifacies* in this study registered complete susceptibility to 5X concentration of DC (1X) of alphacypermethrin and deltamethrin except in district Kanker but registered complete susceptibility to 10X concentrations of both the insecticides in all the three study districts suggesting no change in the ongoing use of pyrethroid for vector control.

In a WHO-coordinated, prospective, observational cohort study to assess the impact of insecticide resistance in malaria vector on the transmission of malaria in district Kondagaon (CG), a congruent district of the present study districts, Kanker and Bastar, the main malaria vector *An*. *culicifacies* registered resistance to deltamethrin in 80 villages in the range of 86–100% in 2013 and registered 13% decrease in median susceptibility from 96% to 83% mortality after the distribution of deltamethrin LLINs and, despite decrease in deltamethrin susceptibility in *An*. *culicifacies*, LLINs were suggested for continued use due to impact on disease transmission [13]. A study was conducted on different biological aspects of *An*. *culicifacies* in some study villages of Kondagaon in 2013 [14]; results of adult susceptibility tests showed decrease in deltamethrin susceptibility after LLIN distribution compared to predistribution period, but was not significant (*p* > 0.05), while the knockdown time values (KdT_50_) showed significant increase (*p* < 0.005) and were also resistant to organophosphate; synergist bioassays with mixed function oxidase (mfo) synergist piperonyl butoxide (PBO), esterase synergists triphenyl phosphate (TPP) and S,S,S-tributyl phosphorotrithioate (DEF), and PBO showed synergism against the major mfo resistance mechanism for pyrethroids, deltamethrin, and alphacypermethrin; TPP and DEF showed synergism against pyrethroids and organophosphate insecticide and indicated involvement of carboxylesterase and nonspecific esterases in conferring pyrethroid resistance as probable minor mechanism; cytogenetic studies indicated prevalence of species B (90%) and C (10%) and were characterized for deltamethrin resistance; genotyping results demonstrated a significant association between *kdr* genotype and deltamethrin phenotype with low frequency (4–5%) mostly in heterozygous condition and play a role in evolving deltamethrin resistance in addition to involvement of mfos and esterases. In another study, *An*. *culicifacies* from different states in India, namely, Gujarat, Chhattisgarh, Haryana, and Rajasthan, *kdr* mutations were identified in low frequency (1.2–7.4%) and mostly in heterozygous condition, and exhibited significant protection against deltamethrin [15]. These studies with *An*. *culicifacies* in CG indicated the propensity for developing intense resistance to pyrethroids that are in use for vector control though the resistance in the presently conducted insecticide intensity bioassays with this species was not high and has not prompted change of insecticide. Resistance intensity assays add more predictive value for making decisions for vector control than other methods such as time-mortality response assays, especially for high insecticide-resistant populations [16]. Three strains of African malaria vector *An*. *funestus* resistant to DC (1X) of deltamethrin (mortality—8 to 24%) showed moderate intensity resistance to 5X (mortality—36 to 89%) and high intensity to 10X (mortality—80 to 100%) the WHO DC (1X) of deltamethrin that had primarily mfo based pyrethroid resistance mechanism [17] and *An*. *gambiae s.l*. in 11 provinces in the Democratic Republic of Congo with evidence of *Kdr* mutations against alphacypermethrin [18]. Though in the Indian major malaria vector *An*. *culicifacies* high level of resistance to pyrethroids is not evidenced so far, but, continued use of pyrethroid insecticides may in coming years render the population resistant to these insecticides and alternative and effective interventions need to be used for managing the resistance. New interventions using chlorfenapyr, a pyrrole class insecticide with novel mode of action (IRAC MoA classification #13-Uncouplers of oxidative phosphorylation via disruption of the proton gradient) [19], rendered DDT-malathion-deltamethrin-bendiocarb-resistant *An*. *culicifacies* from district Raipur (CG) and Panchmahal and Vadodara (Gujarat) and *An*. *stephensi* (Goa) completely susceptible in adult susceptibility tests [20] and were found promising for use in vector control for indoor residual spray [21]. Neonicotinoid class insecticide clothianidin with novel mode of action (IRAC MoA classification # 4A nicotinic acetylcholine receptor (nAChR) competitive modulators) [19] as IRS molecule (SumiSheild 50 WG) was found effective in the management of deltamethrin-resistant *An*. *culicifacies* in phase 2 and 3 evaluation [22, 23] and a mixture of clothianidin and deltamethrin (Fludora Fusion 562.5 WP-SB) for control of deltamethrin-resistant *An*. *culicifacies* in small-scale [24] and large-scale field evaluation [25].

## 5. Conclusion

Intensity bioassays were carried out in three districts Dindori (MP state), and Kanker and Bastar (CG state) exposing *An*. *culicifacies* to 5X and 10X concentrations among pyrethroids, alphacypermethrin, and deltamethrin following WHO guidelines. *An*. *culicifacies* was susceptible in intensity bioassays to 5X concentration of DC of both the insecticides in districts Dindori and Bastar, while in Kanker it was moderate resistant but was susceptible to 10X DC. These results suggest no change in insecticide for vector control. Further, regular intensity resistance monitoring should be carried out in these areas to detect the development of high-intensity resistance in *An*. *culicifacies* and other vectors with provision for proactive decisions on the implementation of effective strategies for the management of insecticide resistance and disease control.

## Figures and Tables

**Table 1:** Intensity bioassay test of *Anopheles culicifacies* against pyrethroid insecticides in district Dindori (Madhya Pradesh state) and districts Kanker and Bastar in Chhattisgarh state in the Year 2022

Districts (State)	Test/ Control	Insecticide/ Control	No. exposed (Replicates)	No. knocked down 1 hr	No. dead 24 hr	% knocked down 1 hr	% mortality 24 hr	Susceptibility status^*∗*^
Dindori (Madhya Pradesh	Test	Alphacypermethrin 0.25% (5X)	100 (5)	98	99	98	99	Susceptible
		Deltamethrin 0.25% (5X)	100 (5)	99	100	99	100	Susceptible
	Control	Pyrethroid	60 (3)	0	0	0	0	NA

Kanker (Chhattisgarh)	Test	Alphacypermethrin 0.25% (5X)	40 (2)	31	37	77.5	92.5	Moderate Resistance
		Alphacypermethrin 0.5% (10X)	20 (1)	18	20	90	100	Susceptible
		Deltamethrin 0.25% (5X)	40 (2)	37	38	92.5	95	Moderate Resistance
		Deltamethrin 0.5% (10X)	20 (1)	20	20	100	100	Susceptible
	Control	Pyrethroid	60 (3)	0	0	0	0	NA

Bastar (Chhattisgarh)	Test	Alphacypermethrin 0.25% (5X)	80 (4)	74	79	92.5	98.8	Susceptible
		Deltamethrin 0.25% (5X)	80 (4)	80	80	100	100	Susceptible
	Control	Pyrethroid	80 (4)	0	2	0	2.5	NA

^*∗*^Susceptible—98-100% mortality; moderate resistance (5X)—<98% mortality.

**Table 2:** Logistic regression of mortality of *An*. *culicifacies* associated with the intensity of bioassay of alphacypermethrin and deltamethrin in districts Dindori (Madhya Pradesh), and Kanker and Baster (Chhattisgarh).

Insecticide	District	Intensity	Odds ratio (95% CI)	
			1 hour	24 hours
Alphacypermethrin	Dindori	1x	Reference	Reference
		5x	79.62(18.60–340.92)^*∗∗∗∗*^	29.33(3.88–221.53)^*∗∗∗*^
		10x	Empty	Empty
	Kanker	1x	Reference	Reference
		5x	6.40 (2.74–14.94)^*∗∗∗∗*^	3.68 (1.04–13.06)^*∗*^
		10x	16.71 (3.66–76.23)^*∗∗∗∗*^	Empty
	Baster	1x	Reference	Reference
		5x	6.28 (2.42–16.28)^*∗∗∗∗*^	16.76 (2.15–130.81)^*∗∗*^
		10x	Empty	Empty

Deltamethrin	Dindori	1x	Reference	Reference
		5x	148.50 (19.93–1106.26)^*∗∗∗∗*^	Empty
		10x	Empty	Empty
	Kanker	1x	Reference	Reference
		5x	15.70 (4.54–54.30)^*∗∗∗∗*^	3.09 (0.67–14.28)
		10x	Empty	Empty
	Baster	1x	Reference	Reference
		5x	Empty	Empty
		10x	Empty	Empty

^*∗*^ *p* < 0.05; ^*∗∗*^*p* < 0.01; ^*∗∗∗*^*p* < 0.001; ^*∗∗∗∗*^*p* < 0.0001.

## Data Availability

All the data used to support the findings of the study are included within the article. The hardcopy of the data can be obtained from the corresponding author upon reasonable request.
